# The Baveno Classification as a Predictor of CPAP Titration Pressure in Obstructive Sleep Apnea Syndrome

**DOI:** 10.3390/arm91060042

**Published:** 2023-12-15

**Authors:** Ahmed Ehab, Axel T. Kempa, Harald Englert, Shaza Almasri Bittar, Aida M. Yousef, Heba Wagih Abdelwahab

**Affiliations:** 1 Pulmonary Medicine Department, Loewenstein Lung Center, 74245 Loewenstein, Germany; axel.kempa@slk-kliniken.de (A.T.K.); harald.englert@slk-kliniken.de (H.E.); shazabittar@hotmail.com (S.A.B.); 2 Chest Medicine Department, Mansoura University, Mansoura 35511, Egypt; aidayousef2020@yahoo.com (A.M.Y.); wagihheba84@gmail.com (H.W.A.)

**Keywords:** obstructive sleep apnea syndrome, apnea–hypopnea index, continuous positive airway pressure, Baveno classification

## Abstract

**Highlights:**

**What are the main findings?**

- After retrospective analysis of 427 patients with OSAS, we found a non-significant correlation between the Baveno classification and the CPAP optimum titration pressure.
- We found a strong positive correlation between the optimal CPAP titration and the severity of OSAS, neck circumference, oxygen desaturation index (ODI), mean oxygen saturation, apnea-hypopnea index (AHI), body mass index (BMI), and cumulative sleep time during periods of SpO_2_ < 90% (T90).

**What is the implication of the main finding?**

- The Baveno classification does not serve as a useful predictor for determining the optimal CPAP titration pressure.
- A higher CPAP titration pressure is significantly predicted by both the ODI and neck circumference as independent factors.

**Abstract:**

Introduction: Obstructive sleep apnea syndrome (OSAS) is a clinical condition characterised by repeated periods of partial or full obstruction of airflow throughout sleep, with impairment of the quality of life and increased mortality with socioeconomic impacts. CPAP therapy is a simple and effective treatment option for OSAS patients. To overcome the clinical and prognostic limitations of AHI—as a sole index of OSAS—the Baveno classification was recently set out and introduced into clinical practice. This study aims to analyse the effect of the Baveno classification on the optimum CPAP titration pressure. Methods: A retrospective analysis of the records of sleep studies in two centres between 2018 and 2021 was carried out. Patients diagnosed with OSAS and recruited for CPAP titration were included. Based on the Baveno classification, the patients were categorised into four groups (A, B, C, and D). Results: Consequently, 700 patients were analysed and 427 patients were included. A significant positive correlation was detected between the CPAP optimum titration pressure and OSAS severity, neck circumference, the oxygen desaturation index (ODI), mean oxygen saturation, the AHI, the BMI, and cumulative sleep time when the SpO_2_ was <90% (T90) on the other side (*p*: <0.0001). A non-significant correlation was seen between the Epworth Sleepiness Scale (ESS), symptom severity, end organ impact, and Baveno classification of the CPAP optimum titration pressure (*p*: 0.8, 0.4, 0.5, and 0.7, respectively). Conclusions: The Baveno classification is not useful in the prediction of CPAP optimum titration pressure. However, the ODI and neck circumference were significant independent predictors of a higher CPAP titration pressure.

## 1. Introduction

Obstructive sleep apnea syndrome (OSAS) is a clinical condition characterised by repeated periods of partial or full obstruction of airflow throughout sleep. It is difficult to estimate the actual prevalence of OSAS, as it is widely underdiagnosed; indeed, 86% to 95% of individuals found in population surveys with clinically significant OSAS report no prior OSAS diagnosis [1]. OSAS is considered to be the most common sleep-related disorder, with a higher tendency in older males. However, the incidence among female patients increases after the menopause [2]. According to epidemiological research, OSAS affects 4% and 2% of middle-aged males and females, respectively [3]. As a consequence, the clinical, social, and even the economic significance of OSAS is well recognised, linking to its prevalence, effect on societal health-related expenses, and patient-related consequences [3]. Many risk factors are linked to the development of OSAS: older age, male sex, obesity, craniofacial and upper airway deformities, and, to a lesser extent, smoking and a family history of snoring or OSAS [4].

Breathing disturbances during sleep, often associated with snoring, are the most obvious measurable markers of OSAS. So, the number of apneas and hypopneas per hour of sleep (the apnea–hypopnea index (AHI)) has become the most used metric to describe the disease and to classify its severity in most guidelines and healthcare standards [1].

The decision to initiate treatment depends upon the severity of the symptoms and/or the presence of any sleep abnormalities, comorbidities, and the occupational status of the patient [5]. The main aim of the OSAS management is to improve the quality of the sleep, with subsequent symptomatic improvement. Untreated moderate to severe OSAS is associated with poor quality of life and increased risk of morbidities (e.g., cardiovascular complications) [6].

Continuous positive airway pressure (CPAP) is the primary treatment for OSAS patients. It lowers the risk of cardiovascular morbidity, such as systemic arterial hypertension, left ventricular systolic dysfunction, and sympathetic nerve activity [7]. The CPAP treatment titration level appears to be essential in CPAP compliance [8]. The results of repeated titrations define the CPAP pressure settings used in the treatment of OSAS. These levels are affected by a variety of anatomical and physiological variations in the upper and lower respiratory tract. The parameters that affect the ideal pressure are not well understood. Certain anthropometric and polysomnographic factors, however, are expected to help in the adjustment of the proper CPAP pressure [9]. Concerns have been raised about the clinical and prognostic importance of the AHI in OSAS because of the disease’s variability and the several OSAS phenotypes. Furthermore, the AHI is a poor predictor of outcomes such as cardiovascular comorbidities or death [5].

To characterise OSAS and guide treatment options, a multicomponent grading system—the Baveno classification—incorporating symptomatology and comorbidities was recently developed [1]. In this study, we analyse the effect of the Baveno classification on the optimum CPAP titration pressure.

## 2. Methods

A retrospective analysis of the sleep records in the Loewenstein Lung Center, Germany, and the Chest Medicine Department of Mansoura University Hospital, Egypt, was carried out. Ethical agreement was acquired from the Institutional Review Board (IRB) at Mansoura University (code number: R.22.09.1804).

Patients who were diagnosed with OSAS between 2018 and 2021 were included in the study. The studies in the sleep laboratory were paused during the first wave of the COVID-19 pandemic between April 2020 and September 2020, in accordance with the local hygiene regulations. This, in turn, affected the total number of included patients.

Patients with (a) a history of upper and lower respiratory tract surgery; (b) nasal diseases (e.g., septal deviation); (c) no indication of CPAP therapy; and (d) patients who refused CPAP treatment were all excluded.

The data from the sleep study results, demographic data, and information on daytime symptoms were retrieved from the medical records.

The polysomnogram results, including the AHI, the oxygen desaturation index (ODI), mean peripheral oxygen saturation (SpO_2_), and cumulative sleep time when SpO_2_ was <90% (T90) throughout sleep, were documented according to the *American Academy of Sleep Medicine* (AASM) *Manual*. [10] The severity of OSAS was categorised as follows: mild for 5 < AHI ≤ 14.9, moderate for 15 ≤ AHI ≤ 29.9, and severe for AHI ≥ 30 [10].

The CPAP titration protocol was applied by polysomnographic technicians. An evaluation of the titration study (involving pressure choice) was performed by a qualified sleep expert.

### 2.1. The Baveno Classification: [1]

**Figure 1:**
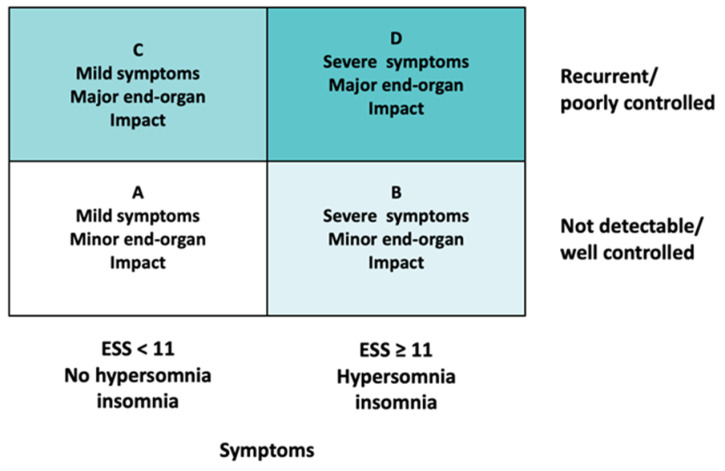
The Baveno classification [3]. ESS: Epworth Sleepiness Scale.

Patients were categorized into four groups (from A to D), according to the Baveno classification [1], depending on their symptoms and the presence of comorbidities. Symptoms included the following: daytime sleepiness, as defined by an Epworth Sleepiness Scale (ESS) score ≥ 11; hypersomnia, as defined by a subjective sleep length ≥ 11 h; and diagnosis of insomnia. The end organ impact was classified depending on the absence or presence of uncontrolled arterial hypertension, atrial fibrillation, heart failure, diabetes mellitus, and a history of stroke (Figure 1).

### 2.2. Statistical Analysis

The data were analysed using SPSS v. 26. Numbers and percentages were used to present nominal variables, while the mean (SD) or median (minimum–maximum) was calculated to present continuous data according to the results of the Shapiro–Wilk testing of the normality of variables. Significance testing was carried out using the chi-square test for ordinal variables, Welch’s *t*-test for parametric variables, and the Mann–Whitney U test for nonparametric variables. Spearman’s correlation was performed for correlation analysis. Linear regression was applied to evaluate the contribution of factors found to be significant in predicting CPAP pressure. The dependent variable was CPAP pressure. The significance level was set at 5%.

## 3. Results

**Figure 2:**
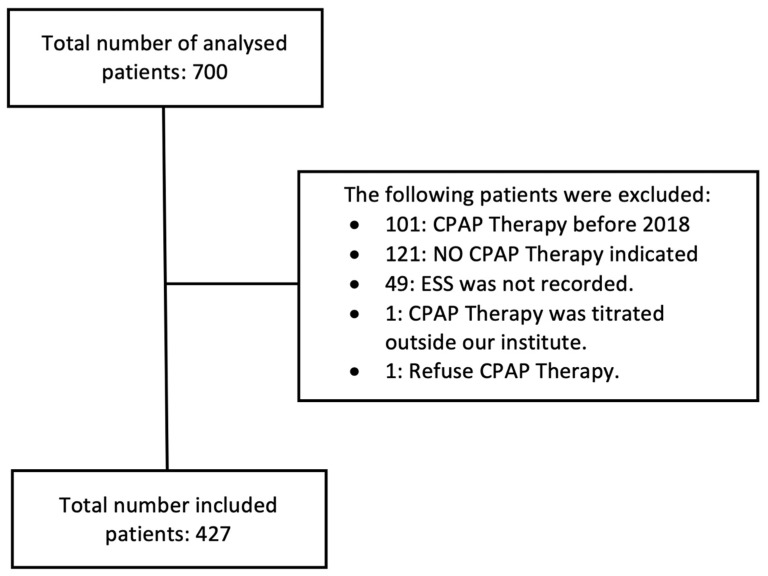
Study chart. CPAP: continuous positive airway pressure, ESS: Epworth Sleepiness Scale.

A total number of 700 patients underwent polysomnography (PSG) between 2018 and early 2021. The collected data were analyzed, and a total number of 273 patients were excluded for the following reasons (Figure 2):

- 101 patients: CPAP therapy was initiated before 2018
- 121 patients: no indication for CPAP therapy
- 49 patients: the ESS was not recorded
- 1 patient: CPAP was titrated outside of the participating institutes
- 1 patient: refused CPAP therapy

Therefore, the total number of included patients was 427 with OSAS diagnosis (mean age 56.1 ± 11.8), 79.9% of whom were males (mean age and BMI of included males were 55.5 ± 11.9 and 32.6 ± 5.3, respectively, and most of them had class B according to the Baveno classification). As shown in Table 1, 55.9% of patients had severe symptoms, and 28.3% were classified as class B, regarding the Baveno classification. The percentage of patients classified as class D was also 28.3%.

The baseline polysomnographic findings are shown in Table 2. The median value (minimum–maximum) of the AHI was 37 (6–109), the median value (minimum–maximum) of T90 was 10 (0–95), the median value (minimum–maximum) of the ODI was 40 (1–128), and the mean SpO_2_ was 90.8% ± 9.5. Most of the cases, 265 (62%), had severe OSAS, 142 (33.3%) patients had moderate OSAS, and 20 (4.7%) patients had mild. The median CPAP optimum titration pressure was 8 cmH_2_O. After CPAP titration, the AHI median (minimum–maximum) dropped to 3 (0–12), the ODI median value (minimum–maximum) was 6 (0–56), the T90 median value (minimum–maximum) was 0 (0–23), and the mean SpO_2_ was improved to 94.4 ± 1.5%.

As shown in Table 3, a significant positive correlation was detected between the CPAP optimum titration pressure on one side and OSAS severity, neck circumference, the ODI, mean SpO_2_, the AHI, the BMI, and T90 on the other side (*p*: <0.0001). In contrast, a nonsignificant correlation was seen between the ESS, symptom severity, end organ impact, and Baveno classification and the CPAP optimum titration pressure (*p*: 0.8, 0.4, 0.5, and 0.7, respectively).

A CPAP optimum titration pressure < 8 and ≥8 cmH_2_O were analysed in the two groups, as showen in Table 4. In patient groups with pressures < 8 and ≥8, no significant differences were found regarding age, sex, the ESS, symptom severity, and end organ impact (*p*: 0.7, 0.3, 0.8, 0.3, and 0.5, respectively). However, for the group with pressure ≥ 8, the BMI, neck circumference, the AHI, T90, and the ODI were significantly higher compared to the group with pressure < 8 (*p*: <0.0001). A nonsignificant difference was detected between the two groups regarding Baveno classification.

As illustrated in Table 5, a linear regression analysis showed that the ODI and neck circumference were significant independent predictors of a higher CPAP titration pressure (*p*: 0.003 and 0.02, respectively).

## 4. Discussion

The Baveno classification was introduced by the working group of the Sleep Disordered Breathing Group of the European Respiratory Society and the European Sleep Research Society to overcome the clinical and prognostic limitations of the AHI as a sole index in OSAS severity [1], as the upper airway obstruction alone does not completely describe the pathophysiology of OSAS. In addition, multiple large studies have consistently demonstrated that the typical phenotype of obesity, male sex, older age, and significant daytime sleepiness reflects just one-quarter of the OSAS patients [11,12]. Therefore, the multicomponent Baveno classification provides the integration of symptoms, including the ESS, as well as cardiometabolic comorbidities in the assessment of OSAS [1].

In this study, we aimed to expand the utility of the Baveno classification to guide the treatment of OSAS in the form of optimizing the CPAP titration pressure. Independent of the AHI, the Baveno classification may guide therapy indication and optimization. The classification may help to avoid unnecessary therapies for individuals with moderate to severe OSAS without symptoms or comorbidities, and it may eliminate the need for medications in people with symptomatic or comorbid OSAS with a low AHI [1].

In this study, we assessed the value of the Baveno classification in the prediction of the optimum CPAP titration pressure. A nonsignificant correlation was seen between the ESS, symptom severity, end organ impact, and the Baveno classification and the CPAP optimum titration pressure. In patient groups with pressures < 8 and ≥8 cmH_2_O, no significant differences were found regarding age, sex, the ESS, symptom severity, end organ impact, and the Baveno classification.

It is well known that it is difficult to establish a fixed formula for an optimal CPAP level [13]. Many worthwhile CPAP pressure prediction models have been developed, however, without a definitive validation [13]. Neither the ESS nor comorbidities were utilised in the models developed by Loredo [14] and by Basoglu [15]. Nadir saturation, the respiratory disturbance index (RDI), and mean saturation were used in the Loredo formula [14], while both neck circumference and the ODI were used in the formula described by Basoglu [15]. Akahoshi et al. [16] developed a prediction equation based on anthropometric, polysomnographic, and cephalometric data in 170 Japanese patients with OSAS. Kırgezen et al. [17] found that increased neck and waist circumferences, and a high hypopharyngeal collapse score, may be predictive for the appropriate CPAP titration pressure in OSAS therapy. In agreement with these results, a significant positive correlation was detected in our study between the CPAP optimum titration pressure on one side and neck circumference and the BMI on the other side. However, linear regression analysis showed that neck circumference was a significant independent predictor of higher CPAP titration pressure.

Additionally, Kırgezen et al. [17] reported a nonsignificant correlation between optimal CPAP pressure, the AHI, and respiratory capacity parameters. In contrast, we found a significant correlation between the optimal CPAP pressure and OSAS severity, the ODI, mean SpO_2_, the AHI, and T90. However, linear regression analysis showed that the ODI was a significant independent predictor of a higher CPAP titration pressure. According to the correlation tests in Günbatar et al. [18], the AHI and the lowest saturation were the two most important predictors of an optimal CPAP level. In fact, the ESS was used only in the formula reported by Lee et al. [19] together with the BMI, minimal SpO_2_, and the RDI. This, to a lesser extent, may explain the absence of a correlation between the Baveno classification and the prediction of CPAP pressure in the titration setting.

Finally, it is worth mentioning that differences in ethnicity and physiology vary between the countries and should be considered in the establishment of a prediction model for CPAP titration pressure [20].

The study’s scope was limited by both the relatively small number of included patients and the retrospective nature of the analysis.

## 5. Conclusions

The Baveno classification is not useful in the prediction of CPAP optimum titration pressure. However, the ODI and neck circumference are significant independent predictors of a higher CPAP titration pressure.

## Figures and Tables

**Table 1:** Characteristics of the studied patients.

	N (427)	%
Age: years (mean ± SD)	56.1 ± 11.8	
Sex		
Males	341	79.9
Females	86	29.1
BMI (mean ± SD)	32.8 ± 5.8	
Neck circumference (mean ± SD)	42.8 ± 4.2	
ESS median (minimum–maximum)	11.5 (0–23)	
Baseline end organ impact		
Minor	219	51.4
Major	208	48.6
Baseline symptoms (n = 417)		
Mild	184	44.1
Severe	233	55.9
Baveno classification (n = 417)		
A	107	25.7
B	118	28.3
C	74	17.7
D	118	28.3

SD: standard deviation, BMI: body mass index, ESS: Epworth Sleepiness Scale.

**Table 2:** Polysomnographic findings including CPAP titration in the studied patients.

	N (427)	%
Initial polysomnography		
AHI median (minimum–maximum)	37 (6–109)	
ODI median (minimum–maximum)	40 (1–128)	
T90 median (minimum–maximum)	10 (0–95)	
Mean SpO_2_ (mean ± SD)	90.8	
OSAS severity		
Mild	20	4.7
Moderate	142	33.3
Severe	265	62
CPAP optimum titration pressure (cmH_2_O) Median (minimum–maximum);	8 (4–13)	
Polysomnography after titration		
AHI median (minimum–maximum)	3 (0–12)	
ODI median (minimum–maximum)	6 (0–56)	
T90 median (minimum–maximum)	0 (0–23)	
Mean SpO_2_ (mean ± SD)	94.4 ± 1.5	

AHI: apnea–hypopnea index, ODI: oxygen desaturation index, T90: cumulative sleep time when SpO_2_ was <90%, OSAS: obstructive sleep apnea syndrome, CPAP: continuous positive airway pressure.

**Table 3:** Correlation of the patients’ parameters with CPAP titration pressure.

	Correlation Coefficient *	Significance
Age	−0.03	0.4
BMI	0.2	<0.0001
ESS	0.01	0.8
Neck circumference	0.31	<0.0001
T90	0.25	<0.0001
AHI	0.35	<0.0001
ODI	0.39	<0.0001
Mean SpO_2_	−0.2	<0.0001
Baveno classification	0.01	0.7
End organ impact	0.03	0.5
Symptom severity	0.04	0.4
OSAS severity	0.3	<0.0001

* Spearman’s rho is used. BMI: body mass index, ESS: Epworth Sleepiness Scale, T90: cumulative sleep time when SpO_2_ was <90%, AHI: apnea–hypopnea index, ODI: oxygen desaturation index, OSAS: obstructive sleep apnea syndrome.

**Table 4:** Association between the patients’ parameters and optimal CPAP titration pressure.

Parameters	Optimum CPAP Pressure < 8	Optimum CPAP Pressure ≥ 8	
Age mean ± SD	56.1 ± 12.2	56.5 ± 11.6	t: −0.2 *p*: 0.7
Sex (n = 409)			
Males	108	218	X^2^ 0.8 p: 0.3
Females	32	51	
BMI mean ± SD	31.1 ± 5.5	33.7 ± 5.7	t: −4.2 *p*: <0.0001
Neck circumference mean ± SD	41.4 ± 3.9	43.4 ± 4.1	t: −4.7 *p*: <0.0001
Mean SpO_2_ mean ± SD	91.3 ± 10.3	90.5 ± 9.3	t: 0.8 *p*: 0.4
AHI median (minimum–maximum)	29 (6–89)	41 (6–109)	Z: −4.8 *p*: <0.0001
T90 (minute) median (minimum–maximum)	6 (0–74)	12 (0–95)	Z: −3.9 *p*: <0.0001
ODI median (minimum–maximum)	30 (1–94)	48 (1–128)	Z: −5.9 *p*: <0.0001
ESS median (minimum–maximum)	12 (1–23)	12 (0–23)	Z: −0.1 *p*: 0.8
Symptom severity (n = 399)			
Mild	66 (47.5%)	109 (41.9%)	X^2^ 1.1 *p*: 0.3
Severe	73 (52.5%)	151 (58.1%)	
End organ impact (n = 408)			
Minor	75 (53.6%)	136 (50.7%)	X^2^ 0.3 *p*: 0.5
Major	65 (46.4%)	132 (49.3%)	
Baveno classification *			
A	40	64	X^2^ 2.3 *p*: 0.1
B	32	80	X^2^ 0.02 *p*: 0.8
C	27	41	X^2^ 0.3 *p*: 0.5
D	40	75	

SD: standard deviation, AHI: apnea–hypopnea index, T90: cumulative sleep time when SpO_2_ was <90%, ODI: oxygen desaturation index, ESS: Epworth Sleepiness Scale. * overall significance X^2^: 3.2 *p*: 0.3.

**Table 5:** Linear regression of independent predictors of optimal CPAP titration pressure.

Predictors	β	*p*-Value
AHI	0.08	0.3
ODI	0.266	0.003
T90	−0.01	0.7
BMI	0.05	0.3
Neck circumference	0.1	0.02
Constant = 4.44, adjusted R^2^ = 0.18, F = 19.1, *p* = ≤0.001		

AHI: apnea–hypopnea index, ODI: oxygen desaturation index, T90: cumulative sleep time when SpO_2_ was <90%, BMI: body mass index.

## Data Availability

Data are contained within the article.
